# Use of Antimalarial in the Management of Fever during a Community Survey in the Kintampo Districts of Ghana

**DOI:** 10.1371/journal.pone.0142106

**Published:** 2015-11-18

**Authors:** Livesy Naafoe Abokyi, Kwaku Poku Asante, Emmanuel Mahama, Stephaney Gyaase, Abubakari Sulemana, Anthony Kwarteng, Jennifer Ayaam, David Dosoo, Dennis Adu-Gyasi, Seeba Amenga Etego, Bernhards Ogutu, Patricia Akweongo, Seth Owusu-Agyei

**Affiliations:** 1 Kintampo Health Research Centre, P. O. Box 200, Kintampo, Brong Ahafo Region; 2 INDEPTH Network, East Legon, P.O. Box KD 213 Kanda, Accra, Ghana; 3 School of Public Health, College of Health Sciences, PO Box LG-13, Legon, Accra, Ghana; Centro de Pesquisa Rene Rachou/Fundação Oswaldo Cruz (Fiocruz-Minas), BRAZIL

## Abstract

**Background:**

Epidemiology of malaria and related fevers in most parts of Africa is changing due to scale up of interventions such as appropriate use of ACTs in the effort towards sustained control and eventual elimination of malaria. The use of ACTs in the management of malaria-associated fever was evaluated in the Kintampo districts of Ghana.

**Methods:**

Household survey was conducted between October 2009 and February, 2011. A random selection of 370 households was generated from 25,000 households existing within the Health and Demographic Surveillance Systems in Kintampo, Ghana at the time. All household members present at the time of survey in the eligible households were interviewed based on a two weeks reported fever recall and the use of antimalarial for the management of fever. A finger-prick blood sample was also obtained from each member of the household present and later examined for malaria parasites using microscopy. Descriptive analysis was performed, with univariate and multivariate analysis used to identify predictors of fever and malaria parasitemia.

**Results:**

A total of 1436 individuals were interviewed from 370 households. Overall, fever prevalence was 23.8% (341/1436) and was 38.8% (77/198) in children < 5 years, 21.3% (264/1238) in older children plus adults. Participants who sought treatment for fever were 84% (285/341) with 47.7% (136/285) using any anti-malarial. Artemisinin-based Combination Therapy use was in 69.1% (94/136) of cases while 30.9% used mono-therapies. Malaria parasitaemia rate was 28.2% (397/1407).

**Conclusion:**

The study reports high community fever prevalence, frequent use of antimalarials for fever treatment and relatively high use of mono-therapies especially in children < 5 years in an area with high malaria parasite prevalence in Ghana.

## Introduction

The decline in malaria transmission, malaria related fevers and consequently mortality due to malaria has been reported in most parts of sub-Saharan Africa [1–8]. This is as a result of availability of more effective malaria control tools, including those used for malaria case management [9–13].

Considerable reduction of fevers associated with *Plasmodium falciparum* from a median of 44% to 22% has been reported over two decades in sub-Saharan Africa [14]. Declining parasite prevalence and malaria mortality have also been reported in Tanzania, Rwanda, Ethiopia Zanzibar and Kenya [3–5, 15]. Although the signs and symptoms of malaria are non-specific, fever is a major symptom for malaria and until recently, fever formed the clinical basis for presumptive treatment of malaria in high malaria transmission areas [16–20].

In line with WHO recommendation for use of ACTs in managing *Plasmodium falciparum* uncomplicated malaria, Ghana changed her anti-malarial drug policy from the use of monotherapies such as chloroquine or sulphadoxine-pyrimethemine (SP) to the current artesunate-amodiaquine (AS-AQ) in 2004 and later included artemether-lumefantrine (AR-LU) and dihydroartemisinin piperaquine (DHP) as alternate first-line treatment [21]. Malaria is a common cause of fever in Ghana as in other malaria endemic countries. Fever in the presence of malaria parasites at the population-level are important indicators for levels of malaria transmission and malaria risks in the communities surveyed. The prevalence of population-level fever and malaria infection have direct impact on malaria case management and use antimalarials. Moreover it was important to assess the use of ACTs in rural populations after five years of the introduction of ACTs.

This study therefore evaluates the use of ACTs for fever as well as fever and malaria parasite prevalence in the Kintampo districts of Ghana. This study was part of a phase IV study of artemisinin combination therapies (ACT) referred to as INDEPTH Effectiveness and Safety Study (INESS) and carried out by the Kintampo Health Research Centre as part of a consortium that evaluated the effectiveness and safety of antimalarials in sub-Saharan Africa.

## Methods

### Study area

The study was carried out in the Kintampo North and South Districts, which are located within the forest-savanna, transitional zone in the Brong Ahafo Region of Ghana. These two districts cover an area of about 7,162 square kms with a residential population of approximately 134,970 within 29,073 households and a median household size of five as at 2009 [22]. The main rainy season is from May to October with an average rainfall of 1250 mm per annum and mean monthly temperature range of 18–38°C. Entomological inoculation rate (EIR) of 231–269 infective bites per person per year [23]. Malaria transmission is perennial with a high annual prevalence of 58% [24]. The study area has two district hospitals, twelve public and private health centres and clinics and thirty community health planning and services (CHPS) compounds. There are also about sixty chemical shops spread within the two districts. Antimalarials are sold over the counter in the two districts as in most parts of Ghana.

### Study design

The study comprised of household surveys conducted between October 2009 and February 2011. The outcome variables of interest were reported fever and treatment sought two weeks preceding interview, the presence of malaria parasitaemia confirmed by malaria microscopy and the use of ACTs in the management of fevers. The interview was performed by study team comprising field workers and investigators using predesigned and tested questionnaires. Demographic (gender and age) and independent variables (level of education, wealth index, residence type) were used to determine predictors of fever and malaria parasitaemia in the studied population.

### Study population and sampling methods

The study was conducted among households randomly selected from the Kintampo Health and Demographic Surveillance System (KHDSS) database, using the resident population as sampling frame. The KHDSS, which is conducted by the Kintampo Health Research Centre (KHRC), covers the entire Kintampo North Municipality (KNM) and the Kintampo South District (KSD). Routine updates of basic health and demographic events of the resident population are core to the KHDSS activities. During the period of this survey, each household was visited three times in a year (once in every four months) to update their basic health and demographic data.

Three hundred and seventy (370) households were randomly selected from a total 25,000 households within the KHDSS. A total of 1436 members interviewed with all resident household members from selected households being interviewed. The sample size was calculated based on a hypothesized average fever prevalence rate of 26% (0.26) given that prevalence in the study area is expected to be higher than the national prevalence of 20% in 2008 ([25]). The expected prevalence of fever in the study area was postulated as 30% (0.30). Using these, a sample size of 1309 was arrived at. A non-response rate of 10% was estimated to arrive at a sample size of 1439. This sample size was calculated for 370 households; however 1436 households based on 4 members per household were eventually interviewed.

### Data collection

Face-to-face interviews were conducted for all household members within each selected household. All respondents consented; adults 18 years and above gave consent on their own behalf and caregivers of minors (<18 years) consented on behalf of minors. Additionally assents were sought from children 12–17years old.

Respondents were asked if they have had fever within two weeks preceding the interview and whether they sought care for the fever episode they reported. Those who sought care were also asked if they took any antimalarials for that fever episode. To facilitate identification of antimalarials used for treatment of this fever episode, a picture catalogue of commonly used antimalarials in the drug shops and pharmacies in the study area was shown to participants. Study team members trained in good laboratory practices, assisted in collecting finger-prick blood samples in Ethylene Diamine Tetra-acetic Acid (EDTA) tubes that were transported to the Kintampo Health Research Centre Clinical laboratory for slide and identification of malaria parasites by microscopy.

### Malaria microscopy

Thin films were fixed with methanol. Both thin and thick films were stained with Giemsa and examined for the presence of malaria parasites using a 100x oil immersion lens. Each smear was independently read by two expert microscopists at KHRC to determine the presence and quantification of malaria parasites. A third microscopist was required to read the smear only if there was discordance between the first two readers on any of the two parameters as described by Swysen and others [26].

### Data management and statistical analysis

All completed forms were checked for consistency and completeness, logged into the computer laboratory of the Kintampo Health Research Centre before double data entry and processing using Visual FoxPro 6.0. Data analysis was done using Stata Corp 11, TX USA. Descriptive statistics were used to generate relative frequencies for demographic data and outcome of interest (fever and parasite prevalence) as well as types of antimalarials used in managing fevers. Both univariate and multivariate logistic regressions were carried out to determine demographic (gender and age) and socio-economic (educational level and wealth index) as predictors of fever and parasitaemia in the population. Odds ratio with 95% confidence interval were used to measure the strength of association at statistical level of *P<0*.*05*. Clustering was adjusted for using the robust standard error estimation.

### Ethical approval

The study protocol and instruments were reviewed and approved by the Ethics Committees of Kintampo Health Research Centre with Federal Wide Assurance (FWA) Number 00011103 and Ghana Health Service with Ethical Clearance ID: GH-ERC:06/5/09. The study team held community meetings with opinion leaders and community members to obtain approval for the study. Written informed consent was obtained from all adult participants, care-givers provided consent for their children and adolescents was also obtained.

## Results

Demographic characteristics of participants, fever and malaria parasite prevalence survey:

Three hundred and seventy households 370 households participated in the study and had household members interviewed and blood sample collected for malaria microscopy. Fever was reported in 20.8% (77) of the households and with malaria parasites being detected in 11.6% (43) of households. In total 1436 individuals from the households participated in the study. About 53.7% (771) were females and 13.8% (198/1436) children < 5 years (Table 1). Of the 1436 participants interviewed, 23.8% (342) reported fever within the preceding two weeks period (Table 1). The prevalence of history of fever among children < 5 years of age and the rest of study population was 38.8% (77/198) and 21.3% (264/1238) respectively. Fever prevalence was relatively higher among males (25.7%) than among females (22.2%).

**Table 1:** Demographic characteristics of 1436 participants, reported fever and parasitaemia infection at household and individual levels.

Demographic Characteristics	No. of persons interviewed n(%)
Households interviewed	370(100)
Households with fever	77(20.8)
Households with parasitaemia	43(11.6)
**Sex**	
Male	665(46.3)
Female	771(53.7)
**Age group**	
< 5yrs	198(13.8)
≥ 5yrs	238(86.2)
**Individuals interviewed**	**1436(100)**
**Individuals who reported fever**	**341(23.8)**
**Individuals who had malaria parasites**	**397(28.2)**
**Parasitaemia among those reporting fever**	**85(24.9)**
* **Proportion of plasmodia that were *P*.*falciparum* infection**	**363(91.4)**
**Rural residents**	**888(61.8)**
**Urban residents**	**548(38.2)**

*29 blood slides were missing

Overall prevalence of malaria parasitaemia in this population was 28.2% (397/1436) (Table 1) with a prevalence of 37.1% (72/194) in children < 5 years and 26.8% (325/1213) in the rest of the study population. Parasitaemia was detected in 24.9% (85/341) of those who reported fever in the preceding two weeks. *Plasmodium falciparum* infection accounted for 91.4% (363/397) of infections and *P*. *malaraie* accounted for 3.3% (13/397). Mixed infections of *P*. *falciparum* and *P*.*malaraie* accounted for 5.3% (21//397) of individuals with malaria parasitaemia.

### Treatment seeking and type of antimalarials used

Majority of the participants who reported fever, 83.6% (285/341) sought treatment (Table 2). However treatment seeking for fever was higher 93.5% (72/77) among children < 5 years of age compared to older children and adults 80.7% (213/264) (Table 2). Among all who sought treatment, 47.7% (136/285) took antimalarials. About 59.7% (43/72) of children <5 years and 43.7% (93/213) of older children and adults took antimalarials (Table 2). Those who used ACTs (artesunate-amodiaquine and artemther-lumifantrine) were 69.1% (94/136) while 30.9% (42/136) used mono-therapies (Table 2). More children < 5 years of age 48.8% (21/43) were given mono-therapies compared with older children and adults, 22.6% (21/93). Mono-therapies used were mainly amodiaquine, artemether, artesunate, chloroquine and sulphadoxine-pyrimethamine.

**Table 2:** Use of antimalarials among participants who sought treatment for fever.

Treatment sought	Total n (%)	< 5 yrs n(%)	≥ 5 yrs n(%)
**Sought treatment**	285 (83.6)	72 (93.5)	213 (80.7)
**Used antimalarial**	136 (47.7)	43 (59.7)	93 (43.7)
**Used recommended ACT**	94 (27.6)	22(28.6)	72 (27.3)
**Used mono-therapies**	42 (12.3)	21 (27.3)	21 (8.0)
**Use of ACT among participants who used antimalarial**	94 (69.1)	22 (51.2)	72 (77.4)
**Artesunate-Amodiaquine**	75 (79.8)	18 (81.8)	57 (79.2)
**Artemether-Lumefantrine**	19 (20.2)	4 (18.2)	15 (20.8)
**Amodiaquine**	21 (50.0)	16 (76.2)	5 (23.8)
**Sulphadoxine-pyrimethamine**	16 (38.1)	2 (9.5)	14 (66.7)
**Other monotherapies**	4 (9.6)	3 (14.3)	1 (4.8)

Less than half of the participants who sought treatment 475 (136/285) took antimalarials. However a higher proportion; 59.7% (43/72) of children < 5 years took an antimalarial compared with 43% (93/213) for older children and adults (Table 2). Among participants who took antimalarial, 69.1% (94/136) used the nationally recommended firstline ACTs which are artesunate-amodiaquine and artemether-lumefantrine. The remaining 30.9% (42/136) used monotherapies which were mainly amodiaquine and sulphadoxine-pyrimethamine. A higher proportion of children < 5 years, 48% (21/43) used monotherapies compared with 22.6% (21/93) of older children and adults. Among those who took antimalarial, a higher proportion of older children and adults (77.4%) took ACTS compared with children < 5 years; 51.2% (22/43) (Table 2).

### Univariate and multivariate predictors of fever and parasitaemia

Both univariate and multivariate logistic regression were used to determine associations between fever, demographic and socio-economic predictors of participants. Only age was significantly associated with the occurrence of fever. Children < 5 years old demonstrated increased risk of fever in both the univariate (OR = 2.35, 95% CI, 1.71–3.22, P<0.001) and multivariate (OR = 2.32, 95% CI, 1.69–3.19, P<0.001) analysis. All the other predictors: gender, educational level of household head, wealth index and place of residence were not significantly associated with the occurrence of fever (Table 3).

**Table 3:** Univariate and multivariate logistic logistic regression for predictors of fever among 341 residents of the Kintampo North Municipality and Kintampo South District.

		Unadjusted odds ratio (univariate)			Adjusted odds (multivariate)		
Characteristics	N (%)	Odds ratio	95% CI	P-value	Odds ratio	95% CI	P-value
**Gender**							
Male	170 (25.6)	1			1		
Female	171 (22.2)	0.83	0.65, 1.06	0.13	0.86	0.67, 1.10	0.22
**Age group**							
≥ 5yrs	264 (21.3)	1			1		
< 5yrs	77 (38.9)	2.35	1.71, 3.22	<0.001	2.32	1.69, 3.19	<0.001
**Educational level of HH**							
None	140 (21.8)	1					
Primary	9 (17.7)	0.77	0.36, 1.61				
Middle/JSS	49 (20.3)	0.91	0.63, 1.31	0.732			
Technical/SSS and above	16 (25.4)	1.22	0.63, 2.36				
**Wealth index**							
Very poor	81 (23.0)	1					
Poor	65 (18.6)	0.77	0.53, 1.11	0.283			
Least poor	79 (22.8)	0.99	0.70, 1.41				
**Residence**							
Rural	199 (22.4)	1		0.13			
Urban	142 (25.9)	1.21	0.95, 1.55				

All the predictors, except gender, were significantly associated with the occurrence of parasitaemia. For age, children < 5 years old demonstrated association with occurrence of malaria parasitaemia by both univariate (OR = 1.61, 95% CI, 1.17–2.22, P = 0.003) and multivariate (OR = 1.63, 95% CI, 1.10–2.24, P = 0.02) analysis (Table 4). Level of education of household heads was significantly associated with presence of parasitaemia when univariate analysis was carried out. Participants whose household heads had primary education were more likely to have malaria infection (49.0%, OR = 2.48, 95% CI, 1.40–4.41) compared with those with no education (Table 4).

**Table 4:** Univariate and multivariate logistic logistic regression for predictors of malaria infection among residents of the Kintampo North Municipality and Kintampo South District.

		Unadjusted odds ratio (univariate)			Adjusted odds (multivariate)		
Characteristics	N (%)	Odds ratio	95% CI	P-value	Odds ratio	95% CI	P-value
**Gender**							
Male	194 (29.9)	1					
Female	203 (26.8)	0.87	0.68, 1.09	0.21			
**Age group**							
≥ 5yrs	325 (26.8)	1			1		
< 5yrs	72 (37.1)	1.61	1.17, 2.22	0.003	1.63	1.10, 2.42	0.02
**Educational level of HH**							
None	179 (28.5)	1					
Primary	25 (50.0)	2.48	1.40, 4.41				
Middle/JSS	58 (24.9)	0.81	0.58, 1.15	0.003			
Technical/SSS and above	13 (20.6)	0.67	0.36, 1.27				
**Wealth index**							
Very poor	141 (39.9)	1			1		
Poor	107 (30.7)	0.66	0.49, 0.91	<0.001	1.72	O.52, 0.99	<0.001
Least poor	49 (14.1)	0.25	0.17, 0.36		0.32	0.20, 0.50	
**Residence**							
Rural	301 (34.4)	1			1		
Urban	96 (18.1)	0.42	0.32, 0.55	<0.001	0.58	0.38, 0.89	0.013

Wealth index was significantly associated with occurrence of malaria infection. When univariate analysis was carried out, participants from both poor households (30.7%, OR = 0.66, 95% CI, 0.49, 0.91, P<0.003) and least poor households (14.1%, OR = 0.25, 95% CI, 0.17, 0.36, P<0.001were less likely to have malaria infection compared with those from very poor (39.9%) households. Also in the multivariate analysis, belonging to a very poor household significantly associated one with malaria infection (P<0.001). The poor households (OR = 0.72, 95% CI, 0.52–0.99) and least poor households (OR = 0.32, 95% CI, 0.20–0.50) were less likely associated with malaria infection (Table 4).

Fewer urban residents (18.1%) had malaria parasites compared with their rural residents (34.4%) and residing in rural areas was significantly associated with occurrence of malaria parasitemia: univariate (OR = 0.42, 95% CI, 0.32–0.55, P<0.001) and multivariate (OR = 0.58, 95% CI, 0.38–0.89, P = 0.013).

## Discussion

The community fever prevalence was 23.8%. Children < 5 years of age had a higher fever prevalence (38.8%); and this was higher than the national prevalence of 19% in a similar age group from the multiple indicator cluster survey [27]. Studies from other parts of Ghana, Nigeria, Kenya and Sierra Leone among others across Africa have also reported lower fever prevalence ranging from 16% to 26% [28, 29] in children < 5 years of age. The higher fever prevalence observed among children in this study could be as a result of the higher burden of malaria in the study area ([24]). High incidence of fever may also result from non-adherence to antimalarial treatment leading to poor treatment outcomes and recurrent fever episodes or the use of ineffective ACTs for malaria episodes. Although adherence to treatment and recrudescence were not investigated in this study, studies carried out in the study area have reported high adherence to ACTs and low recrudescence ([30, 31]). The fever prevalence in children was higher among the rural population compared to their urban counterparts and this can be due to increased risk to infectious pathogens in rural areas.

In this study, age group was the only significant predictor of fever with children < 5 years accounting for most of the fever events compared with older children and adults. Wealth index, educational level and place of residence were not strongly associated with fever events. This finding is contrary to findings from other studies [28, 29, 32] where poverty, low level of education and rural residence significantly influenced the occurrence of fever. The weak association between wealth index, level of education and place of residence with fever could be due to the small numbers involved in the analysis and so this occurrence could be due to chance.

The malaria parasite prevalence in the general population was 28.2%, with prevalence in children < 5 years being 37.1%. This percentage is higher than the national prevalence of 28% reported among children < 5 years [27] but much lower compared with the 58% prevalence reported in 2004 from the same study area seven years prior to this survey [24]. This suggests a decline in malaria parasite prevalence in the study area among this age group but still higher than the national prevalence of 19% ([27]) which is an estimate from across the whole country with different levels of malaria transmission ([33, 34]). The reason could be due to the national scale of the national demographic and health survey which may not have been powered enough to detect difference in parasiteamia. Our study however is more specific or targeted to a smaller geographical area and may be more accurate. Although a decline in parasite prevalence and malaria morbidity has also been reported in numerous studies across Africa [5, 14, 35] due to scale-up of malaria control interventions, certain areas could still have high transmission. The prevalence of malaria parasitaemia among children less than five years of age is usually used as an estimate to determine the burden of malaria in an area [33]. In our study, the prevalence of malaria parasitaemia infection for this age group was high, an indication that malaria transmission is still very high in the area.

This study also demonstrated that only one in four of the participants who reported fever had parasitaemia. This implies that three out of four reported fevers could be related to other conditions rather than malaria. Similar findings where a minority of fevers may be malaria have also been reported [36–39]. This justifies the need for confirmed diagnosis of malaria in health facilities to address over-diagnosis and missed diagnosis and treatment of other causes of fever. Over-diagnosis and subsequent treatment with effective and expensive ACTs is a waste and a drain of resources and invariably increases drug pressure and drug resistance. Parasitological diagnosis of malaria in endemic areas remains important and is recommended [14, 40, 41]. However confirmed parasitological diagnosis requires that microscopy or RDTs are available and used properly by health care providers [42] and this is the only way to ensure rational use of antimalarials.

Age, level of education, wealth index and place of residence were all significantly associated with occurrence of malaria parasitaemia. Children < 5 years of age were more likely to have parasitaemia compared to older children and adults and this could be explained by low immunity status of very young children. Urban dwellers were less likely to have parasitaemia compared to their rural counterparts possibly due to a difference in transmission as well as living in well-screened houses that prevent/reduce mosquito bites. The association of early childhood, rural residence and lower wealth index with parasitaemia is consistent with other studies [29, 40].

In this study, 84% of participants who reported of fever and had sought treatment compared with previously reported rates of 73%–78% among children < 5 years across six sub-Saharan African countries [43]. However, a study in Sudan reported only 39% treatment seeking [32]. The implementation of NHIS in Ghana, which aims at improving financial access to health care could have accounted for prompt care seeking. This must however be interpreted with some caution since information was not obtained by study team on the type or place of care.

Despite the high use of ACTs, this study also reported that one-third of those who received antimalarials used monotherapies which is relatively high at a time when all monotherapies are supposed to have been withdrawn from the market and all health facilities. In a community-based study in 2006, two years after the implementation of ACTs as first-line treatment for antimalarial in the same study area, only 12% to 14% of the respondents used the nationally recommended ACTs for treatment of malaria with 50% still using chloroquine for malaria treatment. In 2006 and 2009, studies in Ghana showed that only 43.0% and 33.0% of patients respectively treated at the health facilities received the nationally recommended ACTs [44] [45].

This study also shows that more children < 5years old received antimalarials compared with older children and adults, however more of the antimalarials received by children < 5 years were monotherapies. This raises some concerns since children are more vulnerable and bare the highest burden of malaria related morbidity and mortality. Availability of more non-tablet peadiatric ACT drug formulations which can be better administered instead of the conventional tablets could improve uptake of ACTs among children ([46]).

The uptake of ACTs could also be influenced by price although this was not investigated in our study. A reduction in the price of ACTs through the affordable medicines facility for malaria, increased the uptake of ACTs (AL) from less than 1% to 42% in Tanzania and from 15% to 42% in Kenya ([47].

This study also reported higher uptake of AS-AQ compared to AL. This could be because AS-AQ was first introduced as firstline treatment for uncomplicated malaria in Ghana in 2004. The policy was later revised to include AL and dihydoartemisinin pepiraquine (DHP) as alternate treatments in 2009 due to reports of adverse drug reactions reported among the public ([21]).

### Conclusion

Although a high proportion of participants in our study used the nationally recommended malaria treatment, intensified efforts need to be in place to increase the uptake of ACTs for the management of malaria and crowd out monotherapies.

Fever and malaria parasite prevalence remains high in Kintampo area with high proportion of fever patients being presumptively treated with antimalarials resulting in malaria overtreatment with one third still using monotherapies. There is the need to enforce the use of ACTs and the withdrawal of monotherapies as well as continual monitoring of compliance to malaria treatment guidelines.

### Limitations

Other causes of fever were not sought and the effect of clustering of participants in households could also lead to biases in malaria parasitaemia prevalence.
